# How Loneliness, Depressed Mood, and Diet Affect Eating Habits and Grocery Shopping

**DOI:** 10.1093/geroni/igaa057.079

**Published:** 2020-12-16

**Authors:** Erica Solway, Dianne Singer, Preeti Malani, Jeffrey Kullgren, Matthias Kirch, Cindy Leung, Julia Wolfson

**Affiliations:** 1 University of Michigan, Ann Arbor, Michigan, United States; 2 University of Michigan Medical School, Ann Arbor, Michigan, United States; 3 University of Michigan School of Public Health, Ann Arbor, Michigan, United States

## Abstract

This study examined the prevalence of loneliness and relationship with cooking and eating habits among older adults using data collected in December 2019 from a nationally representative sample age 50-80 through the National Poll on Healthy Aging. Older adults who live alone were more likely those who lived with others (19% vs. 8%) and those with a high PHQ-2 score were more like than those with a low PHQ-2 score (20% vs. 11%) to report that they do not cook dinner most days (0-2 times/week). Those who rated their diet as fair/poor were more likely to indicate they cook few meals a week (0-2) compared to those who reported a good or very good/excellent diet (18% vs. 11 % vs 8%). Those with a high PHQ-2 score were less likely than those with a low PHQ-2 score to say they do major food shopping less than once a week (31% vs 47%). Those with a high PHQ-2 score were more likely to say that that always ate alone in the last week compared to those with a low PHQ-2 score (17% vs 7%). These findings demonstrate that older adults who lived alone, had a higher PHQ-2 score, and had a poorer diet were more likely to cook and do grocery shopping less often. Strategies and policies to support older adults to address depressive symptoms and to increase cooking and improve diet may have many health and social benefits and will be explored through this session.

